# Effect of JAK Inhibitors on Fatigue and Sleep Quality in Patients with Rheumatoid Arthritis: A Narrative Review

**DOI:** 10.3390/jcm15176559

**Published:** 2026-08-25

**Authors:** Aleksandra Kowalska, Aleksandra Borkowska, Aleksandra Jawoszek, Grzegorz Chmielewski, Łukasz Jaśkiewicz, Magdalena Krajewska-Włodarczyk

**Affiliations:** 1 Department of Mental and Psychosomatic Diseases, School of Medicine, Collegium Medicum, University of Warmia and Mazury in Olsztyn, 10-719 Olsztyn, Poland; al.borkowska14@gmail.com (A.B.); olajawo@gmail.com (A.J.); gchmielewski.gc@gmail.com (G.C.); 2 Department of Human Physiology and Pathophysiology, School of Medicine, Collegium Medicum, University of Warmia and Mazury in Olsztyn, 10-082 Olsztyn, Poland; lukasz.jaskiewicz@uwm.edu.pl

**Keywords:** RA, rheumatoid arthritis, tsDMARD, JAKi, JAK inhibitors, fatigue, sleep quality

## Abstract

Rheumatoid arthritis (RA) is a chronic autoimmune disease in which fatigue and sleep disturbances, in addition to articular symptoms, are of significant importance. These symptoms are among the most common and burdensome complaints reported by patients; they affect their daily functioning and quality of life, and can also exacerbate one another. Currently, the treatment of RA focuses not only on reducing inflammatory activity and preventing structural damage, but also on improving outcomes that matter most to patients. In modern RA treatment, Janus kinase inhibitors (JAKis) are becoming increasingly important; by inhibiting the JAK/STAT pathway, they limit the activity of cytokines involved in the inflammatory response. This review aimed to analyse the effect of selected JAKis on fatigue and sleep quality in patients with RA. An analysis of available study results showed that baricitinib, filgotinib, tofacitinib and upadacitinib reduce fatigue severity. In many cases, improvement was observed as early as the initial stages of treatment and persisted at later follow-up points. At the same time, the extent of this improvement was not uniform and depended on the drug used, the dose, the patient population, and the assessment method adopted. Some analyses also suggested that this effect may have been partly due to reduced pain and improved disease activity. The data on sleep quality are less extensive, but the results suggest that tofacitinib and upadacitinib may also have a beneficial effect in this regard. With tofacitinib, improvements in sleep quality were observed early, affected multiple sleep domains, and, in some studies, persisted over the long term. For upadacitinib, an improvement in sleep quality appeared to be associated with disease control and remission. The smaller number of studies on sleep quality than on fatigue highlights the need to consider this parameter more thoroughly when assessing the efficacy of JAKi treatment and emphasises the importance of a more holistic approach in clinical practice.

## 1. Introduction

Rheumatoid arthritis (RA) is a chronic systemic autoimmune disease of the connective tissue, whose primary clinical manifestation is progressive, symmetrical inflammation of multiple joints, leading to pain, disability, and a significant reduction in health-related quality of life (HRQoL) [1,2]. The deterioration in quality of life in patients with RA results not only from musculoskeletal symptoms but also from extra-articular symptoms, such as fatigue [3]. Although therapeutic goals in RA focus primarily on controlling inflammatory activity and preventing irreversible structural damage, a significant part of the disease burden is specifically attributable to extra-articular manifestations. From the patients’ perspective, systemic symptoms, e.g., chronic fatigue and sleep disturbances, are equally significant and often even more burdensome [4,5]. In view of the above, increasing attention is being paid to the need to consider these aspects in the comprehensive assessment and treatment of patients with RA [4].

Although fatigue is a highly nonspecific symptom, diagnosing it requires a precise distinction between among others, pregnancy and physical exertion. The differential diagnosis of pathological fatigue should encompass neoplastic, musculoskeletal, and autoimmune diseases—with particular emphasis on Sjögren’s syndrome—as well as anaemia of chronic disease, sick building disease and conditions accompanied by fever of unclear aetiology [6,7,8,9,10,11].

Sleep disorders require a careful differential diagnosis because they may be a manifestation of diverse underlying medical conditions. A detailed medical history, including the characterisation of sleep disturbances and identification of comorbid conditions that may impair sleep quality, is essential in the diagnostic evaluation of patients with sleep disorders. Comorbidities associated with impaired sleep quality include cardiovascular and urinary tract diseases, mental health disorders, and chronic pain conditions, such as malignancies and inflammatory rheumatic diseases [12,13].

In patients reporting pain, a comprehensive assessment should include its localization, severity, and circadian pattern. Back pain that intensifies during the second half of the night, leading to awakenings and subsiding after physical activity, suggests an inflammatory nature of the condition and may indicate axial spondyloarthropathy, including ankylosing spondylitis [14]. By contrast widespread pain accompanied by sleep impairment may suggest fibromyalgia, a complex chronic pain disorder characterised by persistent diffuse musculoskeletal pain and heightened pain sensitivity. Fibromyalgia frequently coexists with RA and may independently contribute to widespread pain, non-restorative sleep, daytime sleepiness, and fatigue [15,16,17].

In patients with RA without concomitant fibromyalgia, however, sleep disturbances are most often associated with disease activity, pain and morning stiffness [18,19].

A significant element of modern RA treatment is the introduction of Janus kinase inhibitors (JAKis), which block the Janus kinase/signal transducer and activator of transcription (JAK/STAT) pathway by competitively inhibiting the kinase domain, thus preventing the transmission of intracellular signals from numerous pro-inflammatory cytokines. In RA, this pathway is of crucial importance, as it is responsible for the action of interleukin-6 (IL-6), which promotes lymphocyte activation, and increases the production of interleukin-1β (IL-1β), tumour necrosis factor-α (TNF-α) and interleukin-23 (IL-23), which sustain the T helper 17 cell (Th17) response, as well as interferons (IFN-α, IFN-β, IFN-γ), which intensify the inflammatory response and the expression of chemokines [20,21,22]. Furthermore, JAKis inhibit TNF-α’s secondary effects, which, by inducing IFN-β, amplify the inflammatory cascade [23]. Increasing evidence suggests that the action of JAKis extends beyond controlling disease activity to include improvements in symptoms that are crucial to patients, such as pain and fatigue, and overall physical and mental functioning [24].

## 2. Fatigue and Sleep Disturbance in Patients with RA

Fatigue has been defined as an overwhelming, debilitating and persistent feeling of exhaustion that reduces the ability to function and perform daily activities [25]. Severe fatigue occurs in approximately 41% of patients for whom RA is the only rheumatic disease [26]; however, studies involving all RA patients show that over 70% of them experience, on most days, symptoms similar to those of chronic fatigue syndrome [4]. Fatigue in RA is of a pathological nature, meaning that, unlike physiological exhaustion, it does not resolve with rest. It is considered to be a non-articular symptom, and from 42% to 80% of patients report it as their most burdensome symptom that even surpasses pain [27]. Over a 10-year follow-up of patients with early-stage RA from the ESPOIR cohort, three fatigue trajectories were identified: low, moderate, and high. It was observed that patients remaining on the high fatigue trajectory were more often women, and were characterised by a greater burden of disease symptoms than patients on the moderate or low trajectory. In this group, the following were noted: longer, more severe morning stiffness; a higher Health Assessment Questionnaire Disability Index (HAQ-DI) score; and more tender joints and greater pain severity. In addition, night-time awakenings due to joint symptoms were more frequent, as was the presence of fibromyalgia-like features. At the same time, both physician and patient global assessments on the Visual Analogue Scale (VAS; 0–100) increased, and the subjects more frequently experienced sleep difficulties and increased psychological stress [28,29].

Fatigue and sleep disturbances in RA are closely linked and exacerbate one another. Loppenthin et al. observed, in patients with RA, a linear relationship between sleep disturbances and various dimensions of fatigue, including mental fatigue, physical fatigue and fatigue associated with restricted activity. Importantly, both general and mental fatigue were independently associated with sleep parameters, such as sleep quality, efficiency, duration, latency and daytime functional impairment, emphasising the overlap between the two clinical problems [30].

The results of numerous studies suggest that sleep disturbances affect from 54% to 70% of patients with RA, and the risk of insomnia in this group is almost four times higher than that in the general population [31,32,33]. The most commonly observed sleep disorders include insomnia, fragmented sleep, restless legs syndrome (RLS) and obstructive sleep apnoea (OSA) [34]. In addition, the following are observed: a reduction in total sleep time; a greater number of periods of wakefulness after falling asleep; a higher proportion of non-restorative sleep; and an increase in the number of brief awakenings (mini-arousals) [16,35]. During RA exacerbations, sleep disturbances intensify, manifesting as increased sleep fragmentation, shorter sleep duration, and lower sleep quality [36,37,38]. The identification of risk factors for insomnia, e.g., severe joint pain and stiffness, co-occurring depressive and anxiety disorders, treatment-related side effects, female sex, and long duration of the disease, is crucial for the implementation of appropriately early and targeted therapeutic interventions [34]. However, the severity of sleep disturbances in RA is often linked to the activity of joint inflammation, the severity of pain, and co-occurring mental health disorders, which reflects the complex and multifaceted impact of the disease on sleep quality [39].

## 3. Material and Methods

### 3.1. Literature Search and Study Identification

Our research was conducted by searching through PubMed, Science Direct and Google Scholar. We searched for relevant studies by using the following keywords: “RA”, “rheumatoid arthritis”, “JAKi”, “JAK inhibitors”, “fatigue”, “sleep disturbances”, “sleep disorders”, “baricitinib”, “filgotynib”, “tofacitinib”, “upadacitinib”.

In our analysis, studies from April 2015 to June 2025 were taken into account. We included 26 studies in total. There were 6 studies evaluating the association between baricitinib and fatigue, 2 investigating filgotinib, 4 assessing tofacitinib and 7 analysing upadacitinib. Regarding sleep disturbances 6 studies evaluated the association between tofacitinib and sleep disorders, whereas 1 study investigated the effects of upadacitinib. The search was performed in July 2025 to August 2025.

Among these studies, there were clinical trials, post hoc analyses of randomised controlled trials, real-world evidence studies, patient-reported outcomes studies (PROs) and meta-analyses of randomised controlled trials.

Our study was a literature review based on analysis of previously published studies evaluating the effects of JAK inhibitors on fatigue and sleep outcomes in human populations. Science the study did not involve direct contact with participants, collection of personal data, or any new intervention, approval by an ethics committee was not required.

Because this is a narrative review, no formal systematic review protocol, risk-of-bias assessment, or meta-analysis was performed.

### 3.2. Study Selection and Eligibility Criteria

The review included studies evaluating the effect of JAKis on fatigue and sleep disturbances in patients with RA. The inclusion criteria were the presence of an assessment of fatigue and/or sleep related parameters as an endpoint or one of the clinical parameters analysed, using standardised measurement tools. Publications with the full text available and published in English were included.

### 3.3. Exclusion Criteria

Duplicate publications and reports that did not provide additional relevant information were excluded from the analysis. Non-original publications, including editorials, letters and study protocols, were also excluded. Furthermore, original studies published before 2015 were excluded from the analysis.

Table 1, Table 2 and Table 3 sum up the characteristics and results of the main clinical studies and main real-world studies included in our review.

## 4. Effect of Janus Kinase Inhibitors on Fatigue in Patients with RA

### 4.1. The Pathogenesis of Fatigue in RA, and the Potential Role of JAKis in Fatigue Modulation

Current data suggest that fatigue in RA is not solely a subjective sensation experienced by the patient, but has clear biological correlates in terms of inflammatory markers [25].

In the context of these observations, it is noted that pro-inflammatory cytokines (TNF-α, IL-1β, and IL-6) may initiate the so-called sickness behaviours. These include inter alia, reduced well-being and fever, and a loss of appetite and libido, as well as lethargy, excessive sleepiness, fatigue, low mood, anhedonia and increased sensitivity to pain [40]. This is consistent with research results indicating a strong correlation between the severity of fatigue and higher concentrations of tumour necrosis factor receptor 1 (TNFR1), C-reactive protein (CRP), serum amyloid A (SAA), interleukin-18 (IL-18), IL-6, TNF-α and leptin, while showing an inverse relationship with bone-specific alkaline phosphatase (BALP), immunoglobulin G (IgG) and IFN-γ [25,41,42].

An inflammatory condition that develops peripherally in RA may affect the central nervous system (CNS) via humoral and neural pathways. In the humoral pathway, pro-inflammatory mediators reach the cerebral vasculature and activate microglia; sustained activation of these cells may, over time, weaken the blood–brain barrier (BBB) and increase its permeability. Furthermore, in periventricular organs, where the vessels are fenestrated, the barrier is naturally more permeable, facilitating the penetration of inflammatory mediators, some of which can also enter the CNS via specific transporters. In the neural pathway, pro-inflammatory cytokines interact with afferent fibres and the autonomic nervous system (including the vagus nerve), transmitting pro-inflammatory signals to the solitary tract nucleus that is connected to numerous regions of the brain. Upon initiation of the process in the CNS, microglia and astrocytes are activated, neuroinflammation develops, and the nuclear factor kappa B (NF-κB), JAK/STAT and mitogen-activated protein kinase (MAPK) signalling pathways are activated, which maintains the symptoms [25].

Elevated levels of pro-inflammatory cytokines, such as IFN-α and IFN-γ, also affect tryptophan metabolism and reduce monoamine concentrations. Increased activity of indoleamine 2,3-dioxygenase (IDO) diverts tryptophan metabolism towards the kynurenine pathway, thereby reducing its availability for serotonin synthesis. The resulting kynurenine metabolites, namely 3-hydroxykynurenine and quinolinic acid, may exacerbate oxidative stress, leading to mitochondrial function disorders and neurodegenerative lesions [25,43].

**Figure 1:**
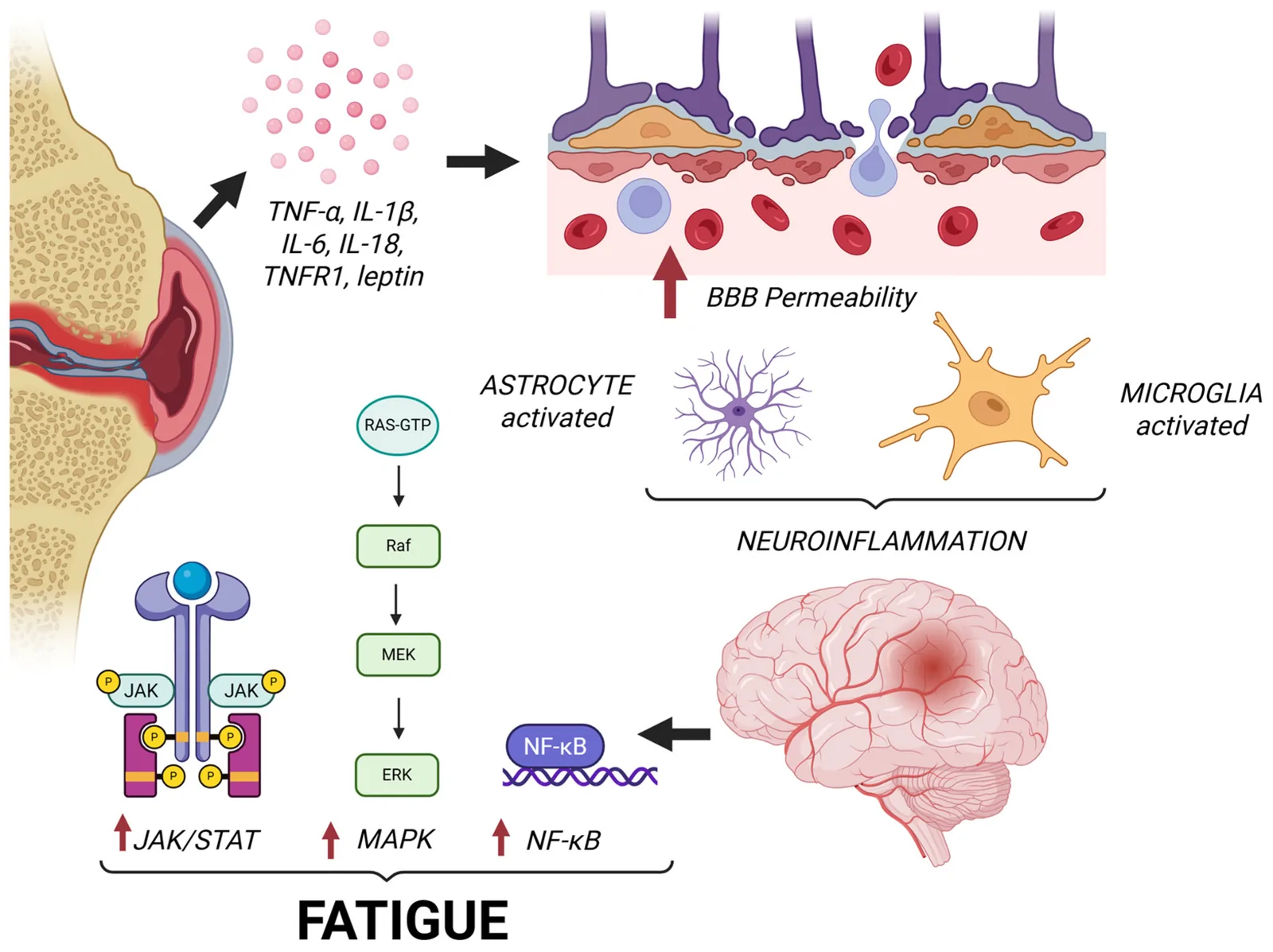
The significance of JAK/STAT pathway activation in the pathophysiology of fatigue. In patients with RA, peripherally developing inflammation leads to increased levels of pro-inflammatory cytokines, such as TNF-α, IL-1β, IL-6, IL-18, TNFR1 and leptin. This is accompanied by the activation of microglia and astrocytes and increased BBB permeability. Consequently, neuroinflammation develops, and signalling pathways, including NF-κB, JAK/STAT, and MAPK, are activated, which intensifies fatigue. List of abbreviations: TNF-α—tumour necrosis factor-α; IL-1β—interleukin 1 beta; IL-6—interleukin 6; IL-18—interleukin 18; TNFR1—tumour necrosis factor receptor 1; BBB—blood–brain barrier; JAK/STAT—Janus kinase/signal transducer and activator of transcription; MAPK—mitogen-activated protein kinase; NF-κB—nuclear factor kappa B. Created in BioRender. Jaskiewicz, L. (2026) https://BioRender.com/mrf5w4y.

The blockade of the JAK/STAT pathway, by targeting the inflammatory processes underlying these mechanisms, may therefore reduce not only pain and disease activity but also influence fatigue, further highlighting its multifactorial nature, which is modulated by metabolic and immunological mechanisms [41]. Figure 1 illustrates the above-described suggested role of the JAK/STAT pathway in the pathophysiology of fatigue.

### 4.2. Assessment of the Effect of Selected Drugs in the JAKi Group on Fatigue in Patients with RA

#### 4.2.1. Baricitinib

In the RA-BALANCE study, conducted in patients with moderately to severely active RA and an inadequate response to MTX, baricitinib 4 mg once daily in combination with MTX significantly improved fatigue compared with placebo plus MTX. FACIT-F scores improved by 9.2 points with baricitinib versus 6.2 points with placebo at week 12 and by 10.0 versus 5.0 points at week 24 (both *p* ≤ 0.001), with the improvement maintained through week 52 (+11.9 points). The proportion of patients achieving the MCID in FACIT-F (≥3.56 points) was also significantly higher with baricitinib at week 12 (64.8% vs. 46.2%; *p* ≤ 0.01) and week 24 (64.1% vs. 26.2%; *p* ≤ 0.001), reaching 80.6% in the baricitinib group at week 52. Improvement in Worst Tiredness was observed as early as week 2 and was maintained though week 52 [44].

A post hoc analysis of the Phase III RA-BUILD and RA-BEACON studies evaluated the ability of baricitinib 2 mg once daily to achieve and maintain clinically meaningful improvements in fatigue in patients with an inadequate response to csDMARDs and bDMARDS, respectively. The MCID in FACIT-F (≥3.56 points) was achieved at week 24 by 59.0% of patients receiving baricitinib versus 42.5% receiving placebo in RA-BUILD (*p* < 0.001; NNT = 6.1) and by 50.0% versus 37.5%, respectively, in RA-BEACON (*p* < 0.05; NNT = 8.0). Among patients who had already achieved the MCID at week 4, the improvement was maintained through week 24 more frequently with baricitinib than with placebo in RA-BUILD (72.1% vs. 57.8%; *p* ≤ 0.05) and numerically more frequently in RA-BEACON (61.5% vs. 52.3%). Similar numerical differences were observed among week 12 responders (74.5% vs. 66.4% in RA-BUILD and 64.9% vs. 54.1% in RA-BEACON), although these comparisons were not statistically significant [45].

The RA-BUILD study assessed baricitinib at doses of 2 mg and 4 mg once daily in patients with RA with an inadequate response or intolerance to csDMARDs. For Worst Tiredness (NRS 0–10), both doses resulted in significant improvement compared with placebo at week 12 (2 mg: *p* = 0.049; 4 mg: *p* = 0.027), with improvement for the 4 mg dose observed as early as day 3 in the daily analysis. In terms of FACIT-F, the 4 mg dose was significantly superior to placebo from week 4, with the effect maintained at subsequent assessment points, including weeks 16, 20, and 24, whereas for the 2 mg dose, statistical significance versus placebo was observed only at week 20. At week 24, the proportion of patients achieving the MCID in FACIT-F (≥3.56 points) was significantly higher with both baricitinib 2 mg (59%) and 4 mg (60%) than with placebo (43%; *p* = 0.001 for both comparisons) [46].

In the RA-BEAM study, fatigue assessed using the FACIT-F was markedly reduced after treatment with baricitinib 4 mg once plus csDMARDs and adalimumab 40 mg every 2 weeks plus csDMARDs. As early as week 4, both therapies were significantly superior to placebo (baricitinib: *p* ≤ 0.001, adalimumab: *p* ≤ 0.01), and the improvement persisted until week 24. Significant differences in favour of baricitinib over adalimumab were noted at week 20 and persisted at weeks 28 and 52 (*p* ≤ 0.05). At week 12, the MCID in FACIT (≥3.56 points) was achieved by 66% of patients receiving baricitinib, 59% receiving placebo, and 68% receiving adalimumab, with significant differences versus placebo for both baricitinib (*p* ≤ 0.05) and adalimumab (*p* ≤ 0.01). At week 52, the proportion of patients achieving the MCID was 60% with baricitinib and 54% with adalimumab, with no statistically significant difference between the groups (*p* = 0.084). In terms of Worst Tiredness, baricitinib showed a significant improvement versus placebo from week 1 and versus adalimumab from week 8 [47].

The RA-BEGIN study demonstrated that baricitinib 4 mg once daily, both as monotherapy and in combination with MTX, was associated with greater improvement in fatigue than MTX alone. Improvement in FACIT-F was observed as early as week 1 (*p* ≤ 0.001 vs. MTX) and was maintained through weeks 24 and 52. At week 24, FACIT-F scores improved by 13.3 points with baricitinib monotherapy and 12.2 points with baricitinib + MTX, compared with 8.9 points with MTX alone (both *p* ≤ 0.001 vs. MTX). The MCID in FACIT-F (≥3.56 points) was achieved by 75% of patients receiving baricitinib monotherapy (*p* ≤ 0.05 vs. MTX), 71% receiving baricitinib + MTX (*p* = 0.268 vs. MTX), and 65% receiving MTX alone. Improvements in Worst Tiredness were also greater with both baricitinib regimens than with MTX from week 1 (*p* ≤ 0.001 for monotherapy and *p* ≤ 0.01 for combination therapy) and were maintained through week 52 [48].

Data from the analysis covering Phase III studies RA-BEAM (MTX-IR) and RA-BEACON biologic disease-modifying antirheumatic drug inadequate responders (bDMARD-IR) show that the reduction in fatigue, as assessed on the FACIT-F during baricitinib treatment, was largely mediated by a reduction in pains (VAS) as well as a decrease in disease activity as measured by the Clinical Disease Activity Index (CDAI). In the MTX inadequate responder (MTX-IR) population, pain reduction accounted for a larger proportion of the effect than improvement in the CDAI score (52% vs. 31%), whereas in the bDMARD-IR population, the contributions of both factors were similar (48% and 48%) [49].

Across the RA-BALANCE study [44], the post hoc analysis of the Phase III RA-BUILD and RA-BEACON trials [45], RA-BUILD [46], RA-BEAM [47], RA-BEGIN [48], and the analysis of the Phase III RA-BEAM (MTX-IR) and RA-BEACON studies [49], baricitinib treatment was associated with improvements in fatigue. In RA-BALANCE [44], the post hoc analysis of RA-BUILD and RA-BEACON [45], RA-BUILD [46], RA-BEAM [47], and RA-BEGIN [48], treatment effects were reported as between-group differences with corresponding 95% confidence intervals, and clinically meaningful improvement in fatigue was evaluated using a predefined minimally clinically important difference in FACIT-F (≥3.56 points). By contrast, in the analysis of RA-BEAM (MTX-IR) and RA-BEACON [49], 95% confidence intervals were not reported and the proportion of patients achieving the MCID for fatigue was not systematically presented, limiting the assessment of the clinical relevance and precision of the observed treatment effects. Furthermore, no direct statistical comparison of the 2 mg and 4 mg doses was performed; therefore, differences in efficacy between the two doses cannot be inferred from comparisons of each dose with its respective control group alone.

#### 4.2.2. Filgotynib

A meta-analysis of the DARWIN 1, DARWIN 2, FINCH 1, and FINCH 2 studies compared filgotinib administered at doses of 200 mg and 100 mg once daily. Both doses significantly improved fatigue, as assessed by the FACIT-F, compared with placebo at week 12 (200 mg: MD 4.76, 95% CI 2.42–7.10, *p* < 0.001; 100 mg: MD 3.60, 95% CI 1.99–5.21, *p* < 0.001), with no significant difference between the two doses. At week 24, the improvement remained significant for filgotinib 200 mg (MD 3.66, 95% CI 1.28–6.04, *p* = 0.003), but not for 100 mg (MD 2.38, 95% CI −0.58–5.34, *p* = 0.12), and the direct comparison significantly favoured the 200 mg dose (MD 1.92, 95% CI 0.86–2.99, *p* < 0.001) [50].

Clinical studies have shown that filgotinib has a rapid and marked effect on fatigue in patients with RA. A post hoc analysis of three randomised Phase III studies (FINCH 1, FINCH 2, FINCH 3) observed a significant improvement, as measured by the FACIT-F, as early as week 4 of treatment. The minimally clinically important difference was defined as an improvement of ≥4 points. The improvement persisted at subsequent assessment points (weeks 12 and 24), and in FINCH 1 and FINCH 3, it also persisted through week 52 [51].

In patients with an inadequate response to MTX (FINCH 1), filgotinib in combination with MTX reduced fatigue more effectively than placebo plus MTX at weeks 4, 12 and 24 (both doses, *p* < 0.001), and numerically greater improvement was observed compared with adalimumab. At week 4, the MCID was achieved by 57.8% and 58.1% of patients receiving filgotinib 200 mg and 100 mg, respectively, compared with 48.0% receiving placebo (*p* = 0.002 for both doses). At week 24, the corresponding proportions were 64.6% and 63.7% versus 52.7% (*p* < 0.001 for both doses) [51].

In patients who did not respond to biologic therapy (FINCH 2), both doses of filgotinib in combination with csDMARDs significantly improved FACIT-F scores compared with placebo + csDMARDs at weeks 4 and 12, with *p* < 0.001 for the 200 mg dose and *p* < 0.01 for the 100 mg dose. At week 24, the significant benefit was maintained for filgotinib 200 mg + csDMARDs (11.5 vs. 6.9; *p* < 0.001). At week 4, the MCID was achieved by 56.3% of patients receiving filgotinib 200 mg + csDMARDs (*p* = 0.013) and 59.7% receiving filgotinib 100 mg (*p* = 0.003), compared 41.7% receiving placebo + csDMARDs [51].

In the population not previously treated with MTX (FINCH 3), all filgotinib regimens showed significantly greater improvement in FACIT-F than MTX monotherapy at week 4 (*p* < 0.001). At this time point, the MCID was achieved by 60.3% of patients receiving filgotinib 200 mg + MTX (*p* < 0.001), 57.6% receiving filgotinib 100 mg + MTX (*p* = 0.025), and 57.6% receiving filgotynib 200 mg monotherapy (*p* = 0.033), compared with 48.4% receiving MTX alone. From week 12 onwards, however, the proportion of patients achieving a clinically significant reduction in fatigue remained similar between groups [51].

In a meta-analysis including the DARWIN 1–2 and FINCH 1–2 studies [50], as well as in a post hoc analysis of the randomised Phase III trials (FINCH 1, FINCH 2, and FINCH 3) [51], statistically significant improvements in FACIT-F scores were observed compared with placebo or the respective control group. In the meta-analysis of DARWIN 1–2 and FINCH 1–2 [50], the results were reported with 95% confidence intervals, whereas these were not provided in the post hoc analysis of FINCH 1, FINCH 2, and FINCH 3 [51]. In the post hoc analysis of FINCH 1, FINCH 2, and FINCH 3 [51] the MCID was defined as an improvement of ≥4 points, and the proportions of patients achieving this threshold were reported. However, in the DARWIN 1–2 and FINCH 1–2 meta-analysis [50], the proportion of patients achieving the minimally clinically important difference in fatigue was not systematically reported. Notably, a direct statistical comparison between the 100 mg and 200 mg doses was performed in the study [50], whereas in the study [51], each dose was assessed separately against the respective control group, without a direct statistician comparison between the two doses.

#### 4.2.3. Tofacitinib

A post hoc analysis of three Phase 3 studies (ORAL Scan, ORAL Standard, ORAL Sync) involved patients with RA and an inadequate response to conventional synthetic disease-modifying antirheumatic drugs treated for 3 months with tofacitinib 5 mg twice daily in combination with a csDMARD—mainly MTX. Compared with placebo, the proportion of patients achieving a normative FACIT-F (≥43.5) was higher (19.9% vs. 12.6%), and a minimally clinically significant improvement in the FACIT-F of ≥4 points was also more common (55.0% vs. 36.1%). At the same time, it was demonstrated that Patient Global Assessment of Disease Activity (PtGA) correlated more strongly with pain than with FACIT-F, suggesting that the patient’s overall assessment is not always an adequate reflection of fatigue severity [52].

Another post hoc analysis of the same three studies (ORAL Scan, ORAL Standard and ORAL Sync) compared the effects of tofacitinib 5 mg and 10 mg twice daily, both administered in combination with csDMARDs, with placebo and, in the ORAL Standard study, adalimumab 40 mg administered subcutaneous every 2 weeks. Both doses of tofacitinib significantly improved FACIT-F scores compared with placebo from month 1, with the benefit maintained through month 6 (*p* < 0.001). The proportion of patients achieving a minimally clinically important improvement in FACIT-F (≥4 points) was also significantly higher with both doses of tofacitinib than with placebo through month 6 (*p* < 0.01). Patients in the placebo group were subsequently switched to active treatment, some at month 3 and the remaining patients after month 6. Compared with adalimumab, tofacitinib 10 mg resulted in significantly greater improvements in FACIT-F at months 1, 3, 6, and 12 (*p* < 0.05), whereas no clear advantage was observed with the 5 mg dose. At month 6, the proportion of patients achieving the FACIT-F MCID was also significantly higher with tofacitinib 10 mg than with adalimumab (*p* < 0.05) [53].

The presence of serological markers, both anti-cyclic citrullinated peptide antibodies (anti-CCP) and the rheumatoid factor (RF), does not appear to significantly modify the reduction in fatigue achieved during treatment. A combined post hoc analysis of 5 Phase III studies (ORAL Step, ORAL Scan, ORAL Solo, ORAL Sync and ORAL Standard) in patients with RA divided subjects into four serological subgroups according to anti-CCP and RF status (positive or negative). At month 3, tofacitinib administered at 5 mg and 10 mg doses twice daily resulted in greater improvement in fatigue, as assessed using the FACIT-F, compared with placebo in all serotypes (*p* ≤ 0.05), suggesting that its beneficial effect on fatigue was observed irrespective of serological status [54].

In contrast, an analysis of data from the CorEvitas registry was conducted, which compared abatacept with tofacitinib in patients with RA who were anti-CCP-positive (titre ≥ 20 U/mL.). Treatment was administered either as monotherapy or in combination with MTX or other non-biologic DMARDs. After 6 months, a numerical advantage in fatigue improvement assessed by patients on a 0–100 scale was observed with abatacept compared with tofacitinib, but the difference was not statistically significant (coef. 1.49; 95% CI −3.17 to 6.15; *p* = 0.531) [55].

For tofacitinib, a post hoc analysis of the Phase III ORAL Scan, ORAL Standard, and ORAL Sync studies [52,53] showed that treatment was associated with statistically significant improvements in fatigue. In addition, a post hoc analysis of the Phase III ORAL Step, ORAL Scan, ORAL Solo, ORAL Sync, and ORAL Standard studies [54], as well as an analysis of data from the CorEvitas registry [55], evaluated the influence of serological status on fatigue outcomes with tofacitinib. Results from the post hoc analysis of ORAL Scan, ORAL Standard, and ORAL Sync [52], the post hoc analysis of ORAL Step, ORAL Scan, ORAL Solo, ORAL Sync, and ORAL Standard [54], and the CorEvitas registry analysis [55] were reported with 95% confidence intervals, whereas 95% confidence intervals were not provided in the other post hoc analysis of ORAL Scan, ORAL Standard, and ORAL Sync [53]. The MCID for fatigue, defined as an improvement of ≥4 points, was reported in the post hoc analyses of ORAL Scan, ORAL Standard, and ORAL Sync [52,53]. In contrast, the proportion of patients achieving the MCID was not systematically reported in the post hoc analysis of ORAL Step, ORAL Scan, ORAL Solo, ORAL Sync, and ORAL Standard [54] or in the CorEvitas registry analysis [55]. In addition, no direct statistical comparison between the 5 mg and 10 mg doses was performed; each dose was assessed separately against the respective control group.

#### 4.2.4. Upadacitinib

In the ENDAVOUR study, upadacitinib used both as monotherapy and in combination treatment with csDMARDs reduced perceived fatigue. In half of the patients, a clinically significant improvement on the Fatigue VAS (MCID: −1.12 points) was achieved within a median of 7 days of treatment initiation (95% CI 5–14), whereas with the FACIT-F, the median time to achieve the MCID (≥4.0 points) was 58 days (95% CI 29-NA). The improvement in Fatigue VAS was maintained though 6 months, with a mean reduction of 17.9 points at month 6 [56].

The effect of upadacitinib on fatigue in the SELECT-NEXT study, conducted in patients with RA receiving background csDMARD therapy (most commonly regimens including MTX) and with an inadequate response to csDMARDs, was similar for both doses. Significant improvement in FACIT-F was already observed at week 4 and was maintained through week 12 for both doses (*p* < 0.001 vs. placebo). At week 12, both 15 mg (+7.91 points on FACIT-F) and 30 mg (+7.74 points on FACIT-F) were associated with a statistically significant improvement compared with placebo (+2.96 points), and the difference between the doses was minimal (≈0.2 points). At week 12, a minimally clinically important improvement in FACIT-F (MCID ≥ 4.0 points) was achieved by 64% of patients receiving upadacitinib 15 mg (*p* < 0.001) and 57% receiving 30 mg (*p* < 0.01) compared with 41% receiving placebo [57].

A post hoc analysis of the SELECT-CHOICE study, which compared upadacitinib at 15 mg, administered orally once daily, with abatacept administered intravenously according to body weight, with background csDMARD therapy, showed a reduction in fatigue, as assessed using the FACIT-F, in both groups. At week 12, the FACIT-F score increased by 9.6 points (95% CI 8.3–10.9) with upadacitinib and by 8.4 points (95% CI 7.1–9.6) with abatacept, and at week 24, it increased by 10.7 points (95% CI 9.4–12.1) and 10.3 points (95% CI 9.0–11.7) respectively. A minimally clinically important improvement in FACIT-F (MCID ≥ 4 points) was achieved by 68% of patients receiving upadacitinib and 62% receiving abatacept at week 12, and by 67% and 65%, respectively, at week 24. The differences between the treatments were numerically small and not statistically significant, suggesting no clear advantage of either treatment in terms of fatigue improvement [58].

In the extended phase of the study, after week 24, all patients received upadacitinib 15 mg orally once daily, and patients who had previously been treated with abatacept were switched to this drug. In week 216, a reduction in fatigue, as assessed using the FACIT-F, continued to be observed (an increase of 11.0 points in patients continuing treatment with upadacitinib and 10.9 points after switching from abatacept to upadacitinib), indicating the durability of the therapeutic effect in this respect [59].

An analysis of data from the CorEvitas registry assessed adult patients with RA having moderate/high disease activity (CDAI score > 10) who were initiating treatment with upadacitinib. Six- and 12-month follow-up data were used, with 76% of the patients included in the 12-month analysis having previously been included in the 6-month analysis. Fatigue was assessed as a patient-reported outcome on the 0–100 VAS (a mean of 57.9 ± 26.8 at baseline), with a greater improvement observed after 12 months than after 6 months (mean change: −9.8 points after 6 months {*p* ≤ 0.001} and −12.4 points after 12 months {*p* ≤ 0.001}). The reduction was more evident in patients who continued treatment with upadacitinib until the follow-up visit (mean change: −13.1 points after 6 months and −17.0 points after 12 months; both *p* ≤ 0.001). At 6 months, 42.0% of patients achieved the MCID, defined as a reduction of ≥10 points, with a numerically higher proportion observed at 12 months. In subgroup analyses, patients previously exposed to TNF inhibitors (TNFi) showed a numerically smaller improvement in fatigue than those receiving upadacitinib as first-line treatment (month 6: −7.7 vs. −13.1 points; months 12: −8.2 vs. −20.4 points; all within-group changes *p* ≤ 0.001), suggesting a less pronounced improvement in patients previously exposed to TNFi [60].

A comparative study involving a switch from the previously used TNFi to upadacitinib, another TNFi, or a different mechanism of action (IL-6 inhibitor, cluster of differentiation 20 (CD20) inhibitor, or cytotoxic T-lymphocyte-associated protein 4 (CTLA-4) co-stimulation modulator) assessed fatigue using a four-point physician-reported scale (none, mild, moderate, or severe). After at least 6 months of treatment, absence of fatigue was reported more frequently in patients who switched to upadacitinib than in those who switched to another TNFi or to a therapy with a different mechanism of action. Although these differences did not reach statistical significance, a numerical trend favouring upadacitinib was observed (*p* = 0.055 and *p* = 0.075) [61].

The SELECT-COMPARE study, which compared adalimumab 40 mg every 2 weeks, upadacitinib 15 mg once daily, and placebo in patients with active RA who has an inadequate response to MTX, assessed fatigue using the FACIT-F. After 12 weeks, the mean improvement in the FACIT-F score was greater in the upadacitinib-receiving group than in the placebo- and adalimumab-receiving groups (+8.95 vs. +4.81 and +7.44), and a clinically significant improvements (≥4 points) was achieved by 64%, 46% and 62% of patients, respectively. In subsequent follow-up, upon approval of rescue therapy (switching treatment in patients with an inadequate response) and the switch from placebo to upadacitinib in week 26, long-term comparisons focused on upadacitinib and adalimumab. In weeks 26 and 48, upadacitinib continued to show superior efficacy in terms of fatigue as assessed using FACIT-F (mean change of +9.67 vs. +8.24, and +10.23 vs. +8.93, respectively) [62].

Across clinical trials and real-world analyses, upadacitinib was consistently associated with improvements in fatigue, as demonstrated in the ENDEAVOUR study [56], SELECT-NEXT [57], a post hoc analysis of the SELECT-CHOICE and its long-term extension [58,59], the CorEvitas registry analysis [60], the comparative analysis by Caporali et al. [61], and SELECT-COMPARE [62]. Between-group differences were reported with 95% confidence intervals in the ENDEAVOUR study [56], SELECT-NEXT [57], a post hoc analysis of the SELECT-CHOICE and its long-term extension [58,59], the CorEvitas registry analysis [60], and SELECT-COMPARE [62], whereas 95% confidence intervals were not reported in the comparative analysis by Caporali et al. [61]. The minimally clinically important difference for fatigue was defined and assessed in the ENDEAVOUR study [56], SELECT-NEXT [57], a post hoc analysis of the SELECT-CHOICE [58], the CorEvitas registry analysis [60], and SELECT-COMPARE [62]. In contrast, the proportion of patients achieving the MCID was not systematically reported in the long-term extension of SELECT-CHOICE [59] or in the comparative analysis by Caporali et al. [61]. In addition, where both the 15 mg and 30 mg doses were evaluated, no direct statistical comparison between the two doses was performed; each dose was assessed separately against the respective control group.

## 5. Effect of JAKis on Sleep Quality in Patients with RA

### 5.1. The Pathogenesis of Sleep Disturbances in RA and the Potential Role of JAKis in Modulating Them

In the pathogenesis of sleep disturbances in RA, a two-directional relationship is observed: increased disease activity impairs sleep quality, while sleep disturbances may exacerbate the inflammatory process and contribute to the aggravation of RA symptoms [63]. In RA, elevated levels of pro-inflammatory cytokines, such as IL-6 and TNF-α, are observed, which adversely affect normal sleep architecture. At the same time, sleep deprivation is associated with increased levels of IL-6 and TNF-α, disruption of helper T cell balance, reduced natural killer cell activity, and dysfunction of the hypothalamic–pituitary–adrenal axis. Consequently, inflammation in the synovial membrane is sustained, and disease activity is exacerbated, indicating a vicious circle between inflammation and sleep disturbances [64].

The pathophysiology of sleep disturbances in patients with RA, involving the JAK/STAT pathway, remains unclear. However, available data from animal model studies suggest that JAK/STAT signalling in the central nervous system may regulate inflammation-associated sleep. One study conducted on insects suggests that the JAK/STAT pathway is a key component of the gut–brain axis, in which oxidative stress or intestinal inflammation leads to the release of IL-6-like cytokines (e.g., Unpaired 2/3, Upd2/3) from enteroendocrine cells. These cytokines penetrate the central nervous system and activate the JAK/STAT pathway in the glial cells of the BBB, thus affecting sleep regulation. Under physiological conditions, wakefulness is supported by neuropeptides, such as allatostatin A (AstA), whereas in inflammatory conditions, JAK/STAT activation in BBB glia reduces AstA receptor (AstA-R1 and AstA-R2) expression, thereby weakening signals that promote wakefulness. Consequently, increased drowsiness occurs, which is perceived as an adaptive mechanism that promotes the body’s recovery during an inflammatory response [65]. From a clinical perspective, it may therefore be assumed that JAKis, by reducing JAK/STAT pathway activity and lowering pro-inflammatory cytokine levels, may improve sleep quality in patients with RA. By blocking IL-6 signalling to the central nervous system, JAKis can reduce the neuroimmunological mechanisms underlying the so-called “sickness-induced sleep”, thereby alleviating fatigue and sleep disturbances associated with chronic inflammation.

**Figure 2:**
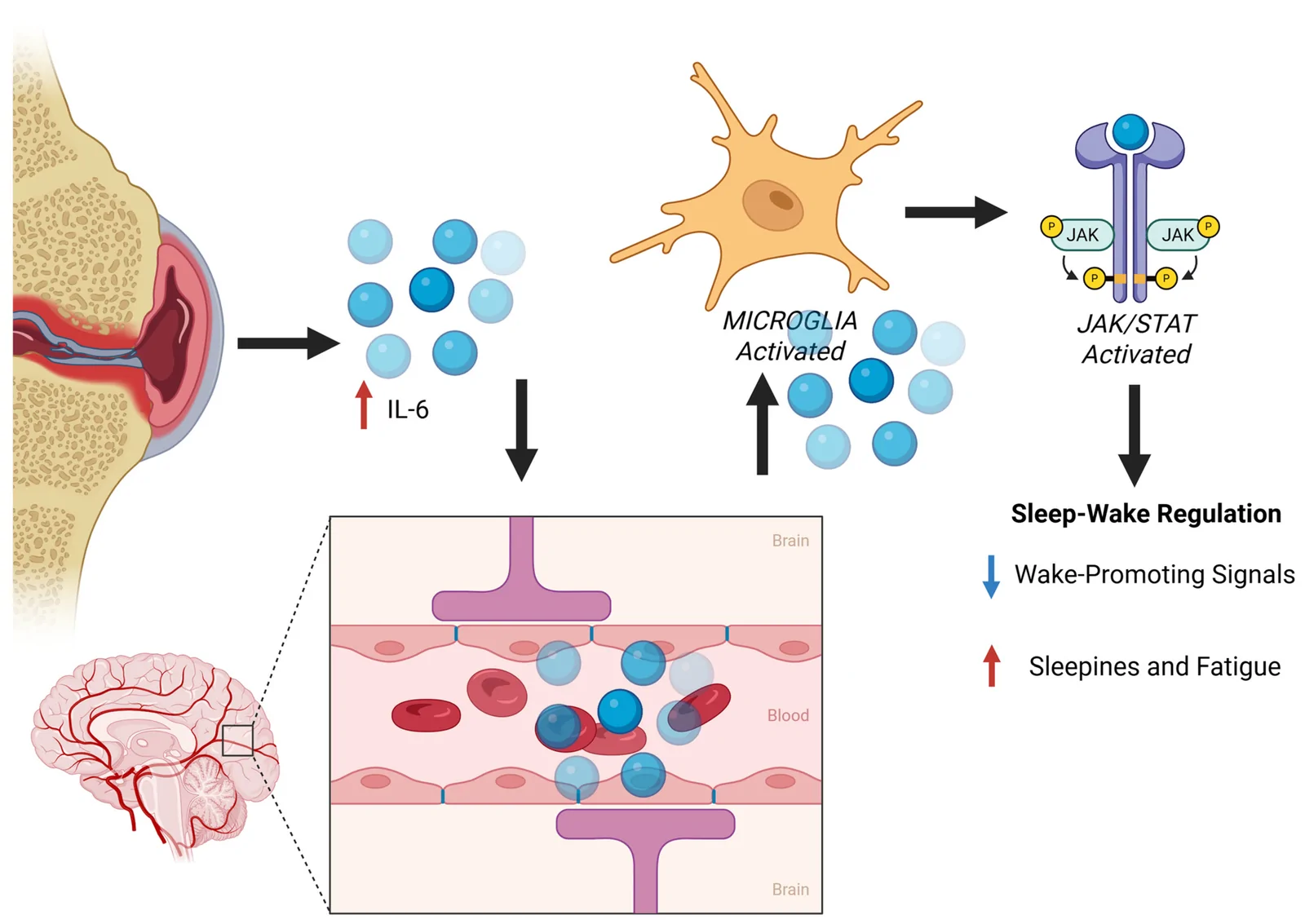
The significance of JAK/STAT pathway activation in the pathophysiology of sleep disturbances. In patients with RA, IL-6 production is increased, which penetrates the central nervous system and activates the JAK/STAT pathway in glial cells of the BBB. As a result, the signals that promote wakefulness are weakened, while drowsiness and fatigue are intensified. List of abbreviations: IL-6—interleukin 6; JAK/STAT—Janus kinase/signal transducer and activator of transcription; BBB—blood–brain barrier. Created in BioRender. Jaskiewicz, L. (2026) https://BioRender.com/wkpgbch.

Figure 2 illustrates the above-described suggested involvement of the JAK/STAT pathway in the pathophysiology of sleep disturbances.

### 5.2. Assessment of the Effect of Selected Drugs in the JAKi Group on Sleep Quality in Patients with RA

#### 5.2.1. Tofacitinib

A post hoc analysis of aggregate data from three randomised Phase III studies, namely ORAL Scan, ORAL Sync and ORAL Standard [66,67,68], showed that tofacitinib may improve sleep quality as assessed using the Medical Outcomes Study Sleep Scale (MOS-SS). Tofacitinib at doses of 5 mg and 10 mg twice daily, combined with a csDMARD, primarily MTX, led to a statistically significant improvement in the MOS-SS Index I and II as early as the first month of treatment compared with placebo, and this effect persisted through month 6. After the first month of treatment with tofacitinib, improvements were noted in sleep adequacy, sleep disturbance, sleep quantity, and somnolence. The analysis also showed that the use of tofacitinib at both doses led to a statistically greater improvement in terms of sleep problems, compared with adalimumab, as evidenced in months 1, 3 and 6 of the treatment according to the MOS-SS Index I and II. Furthermore, the 10 mg dose of tofacitinib provided a significant advantage over adalimumab at the 12-month follow-up, demonstrating significantly greater improvements in selected sleep parameters, including sleep adequacy, sleep disturbance, sleep quantity, and somnolence [53].

In contrast, a single post hoc analysis of data from the ORAL Sync study, comparing tofacitinib with placebo in combination therapy with csDMARDs, showed that both the 5 mg dose and 10 mg twice daily led to a significant improvement in sleep quality, subjectively assessed by patients as early as 3 months after treatment, with the effect persisting until month 12. The proportion of patients achieving at least a minimal clinically meaningful improvement was significantly higher in the tofacitinib-receiving groups than in the placebo group. The improvement in sleep quality was part of a broader clinical benefit, which also included reduced pain and fatigue [69].

The Phase III ORAL Scan study assessed the long-term effect of tofacitinib at doses of 5 mg or 10 mg twice daily, in combination treatment with MTX, on sleep quality in patients with RA (n = 797). It was shown that the use of tofacitinib at both doses was associated with a significant improvement in sleep parameters as early as 3 months after treatment (least-squares mean (LSM) change of −5.4 for the 5 mg dose, and −5.5 for the 10 mg dose, respectively, compared with −2.5 for placebo + MTX; *p* < 0.05). This improvement was maintained throughout the full 24-month follow-up period, and patients who initially received placebo after switching to tofacitinib achieved a comparable improvement in sleep quality to that in patients treated with tofacitinib from the beginning of the study [70].

In contrast, a Phase III clinical study by Strand et al., involving a smaller group of patients (*N* = 399) and conducted in patients with an inadequate response to TNFi, assessed the efficacy of tofacitinib in combination with MTX. Analysis of patient-reported outcomes after 3 months of treatment showed no statistically significant improvement in sleep quality, as measured by the MOS-SS, in the tofacitinib plus MTX group compared with the placebo plus MTX group. The mean change from baseline was −6.80 for the 5 mg dose twice daily in combination with MTX and −5.82 for the 10 mg dose twice daily in combination with MTX, while the control group (placebo in combination with MTX) showed an improvement of −3.80 points [71].

The ORAL Solo study confirmed that tofacitinib at a dose of 10 mg twice daily as monotherapy significantly improves sleep quality. After 3 months of treatment, patients receiving this dose reported an improvement of 10.18 points on the MOS-SS, whereas in the placebo group, improvement was only 4.81 points. The 5 mg dose twice daily brought about a smaller improvement (7.13 points) that was not statistically significant. After 6 months of treatment, the positive effect persisted in all patients, with a reduction in sleep disturbances of almost 10 points for the higher dose of tofacitinib, and approximately 7.5 points for the lower dose [72].

In contrast, the results of the 24-month ORAL Start study showed that monotherapy with tofacitinib 5 mg twice daily led to a significant improvement in sleep quality on the MOS-SS compared with MTX. Statistical significance was confirmed in month 3 (LSM: −12.60 vs. −9.11; *p* < 0.05) and in month 12 of the observation (LSM: −12.70 vs. −8.47; *p* < 0.05). Although positive trends were observed with the 10 mg dose, no statistical superiority over MTX was demonstrated, indicating the need for more in-depth analysis of the dynamics of the drug response, depending on the dosage and the duration of follow-up [73].

Across a post hoc analysis of ORAL Scan, ORAL Sync, and ORAL Standard [53], a post hoc analysis of ORAL Sync [69], the Phase III ORAL Scan study [70], ORAL Solo [72], and ORAL Start [73], tofacitinib treatment was associated with statistically significant improvements in sleep outcomes assessed using the MOSS-SS. In contrast, the Phase III study reported by Strand et al. [71] did not demonstrate a statistically significant improvement in sleep. Between-group treatment effects with corresponding 95% confidence intervals were reported in ORAL Scan [70], the study by Strand et al. [71], and ORAL Solo [72], whereas 95% confidence intervals were not provided in the post hoc analysis of ORAL Scan, ORAL Sync, and ORAL Standard [53], the post hoc analysis of ORAL Sync [69] or ORAL Start [73]. Across these studies, the proportion of patients achieving a minimally clinically important difference in sleep outcomes was not systematically reported. Furthermore, no direct statistical comparison between the 5 mg and 10 mg doses was performed; each dose was evaluated separately against the respective control group, precluding conclusions regarding statistically significant differences in efficacy between the two doses.

#### 5.2.2. Upadacitinib

In the SELECT-BEYOND study, the effect of upadacitinib on sleep disturbances was evaluated in patients with RA and an inadequate response to bDMARDs. Sleep-related outcomes were assessed using the Insomnia Severity Index (ISI), scored from 0 to 28, with increasing scores reflecting greater insomnia severity. The minimally clinically important difference for ISI was defined as a decrease of ≥8.4 points, corresponding to a moderate improvement. A statistically significant difference in the proportion of patients achieving an improvement ≥ MCID in ISI at week 12 was observed only with the 30 mg dose of upadacitinib compared with placebo. After 12 weeks of treatment, upadacitinib 30 mg resulted in a significantly greater improvement in ISI compared with placebo (LSM change: −3.32 vs. −1.69; *p* < 0.01), whereas the improvement observed with the 15 mg dose (−2.53 vs. −1.69) did not reach statistical significance. The proportion of patients achieving a value equal to or higher than the normative value on the ISI scale (0–7) was also greater with 30 mg of upadacitinib than with placebo (44% vs. 33%; *p* < 0.05). Furthermore, the improvement in ISI was accompanied by simultaneous improvements in other key patient-reported indicators, including the PtGA, pain severity, and the HAQ-DI. The results of the study strongly suggest that the observed improvement in sleep quality may be a secondary effect of effective RA activity control [74].

In the SELECT-BEYOND study [74], between-group treatment effects were reported with corresponding 95% confidence intervals, and the minimally clinically important difference was incorporated into the assessment of clinically meaningful responses. No direct statistical comparison between the 15 mg and 30 mg doses was performed; instead, each dose was evaluated separately against the respective control group.

There are reports suggesting a potentially beneficial effect of upadacitinib 15 mg on sleep parameters in patients with RA. One such report is the SLEERA study, which assessed both subjective sleep quality and objective actigraphy-derived parameters. After 3 months of treatment with upadacitinib 15 mg once daily, a significant improvement in subjective sleep quality was observed, with the mean Pittsburgh Sleep Quality Index (PSQI) score decreasing by 2.26 points (*p* < 0.001) and the proportion of patients with clinically significant sleep disturbances (PSQI >5) decreasing from 76% to 55%. A favourable trend was also observed in objective measures, with the proportion of patients with low sleep efficiency (<85%) decreasing from 51% at baseline to 38% after 3 months. The greatest improvements were observed in patients who achieved both clinical remission and remission as assessed by DAS28-CRP. Thus, the findings of the SLEERA study suggest that upadacitinib 15 mg may have a beneficial effect on sleep quality in patients with RA. However, given the small study population, these observations should be regarded as preliminary and require confirmation in studies involving larger patient populations [75].

## 6. Clinical Use and Safety Profile of JAK Inhibitors

Current ACR 2021 and EULAR 2025 recommendations indicate that, in patients who fail to achieve the predefined treatment target despite therapy with csDMARDs, one of the available options for treatment escalation is the addition of JAKis to ongoing csDMARD therapy [76,77]. At the same time, in patients who cannot use csDMARDs as comedication, JAK inhibitors, similarly to IL-6 pathway inhibitors, may offer certain advantages over other bDMARDs, supporting their use without concomitant csDMARD therapy [77].

Before initiating JAK inhibitor therapy, a comprehensive assessment of the patient’s individual risk profile should be performed. Particular consideration should be given to age over 65 years, a history of smoking, and the presence of cardiovascular risk factors, including hypertension, diabetes mellitus, and obesity. A history of malignancy and factors associated with an increased risk of thrombotic events, coagulation disorders, immobilisation, and the use of hormonal therapy [77].

An additional component of the assessment prior to treatment initiation is the evaluation of its potential adverse effects. The most common adverse events include infections, with particular attention to herpes zoster reactivation and the risk of serious infections [78,79]. In addition to infectious complications, the use of JAK inhibitors is associated with a risk of thromboembolic events and malignancies, particularly malignant neoplasms of the skin and respiratory system. Attention should also be given to ruxolitinib, which, compared with other JAK inhibitors, has been associated with a higher proportion of fatal outcomes [78].

Considering current treatment guidelines, the need for appropriate patient selection, the assessment of individual risk factors, the adverse event profile, and the availability of alternative therapeutic options, the effects of JAK inhibitors on fatigue and sleep disturbances should be considered as part of a broader evaluation of the clinical benefits associated with therapy. Improvement in these symptoms may be clinically meaningful and may contribute to better quality of life; however, it should not, by itself, constitute a decisive criterion for treatment selection. The final decision to initiate JAK inhibitor therapy should be individualised, taking into account the overall benefit–risk balance and following discussion with the patient regarding the available therapeutic options.

## 7. Limitations

Several important limitations of the available data should be considered when interpreting the findings of the review. According to current recommendations, the preferred treatment regimen is the use of JAK inhibitors in combination with csDMARDs, a pattern mirrored in the analysed studies where patients routinely received concurrent therapies from multiple drug classes. Combination therapy may act as a confounding factor, making it challenging to attribute the observed clinical effects exclusively to JAK inhibitors and constraining the precise evaluation of their independent contribution to the study endpoints.

Another important limitation is the difficulty in determining whether the observed improvements in fatigue and sleep are directly attributable to JAK inhibition. These outcomes may partly reflect an overall reduction in disease activity, inflammation, pain, and other RA-related symptoms, making it difficult to distinguish a specific effect of JAK inhibitors on fatigue and sleep from the broader clinical response to treatment. Moreover, both fatigue and sleep disturbances are multifactorial and may be influenced by comorbid conditions and other patient-related factors, including depression, anxiety, anaemia, fibromyalgia, and primary sleep disorders. These factors may therefore introduce additional potential confounding when interpreting treatment-related changes in fatigue and sleep. Therefore, the mechanisms underlying the effects of JAK inhibitors on these outcomes remain unclear and require further investigation.

Additionally, the research base for sleep outcomes is substantially smaller than the literature on fatigue. Because available trials centred exclusively on tofacitinib and upadacitinib, one cannot assume these sleep outcomes apply to the JAK inhibitor class as a whole.

Another limitation is the considerable heterogeneity of the study populations, including differences in patients’ clinical characteristics, the size of the study groups, and treatment regimens. In some studies, the size of individual groups was small or markedly uneven, which may limit the precision of treatment effect estimates and the reliability of comparisons. Additionally, different tools and scales were used in individual studies to assess fatigue and sleep quality, which limits the direct comparability of the results obtained. Because these scales measure clinical symptoms rather than direct pharmacological activity, isolating the true drug-induced response from non-pharmacological influences remains challenging.

It should also be noted that some of the available data came from post hoc analyses of clinical trials in which fatigue or sleep-related parameters were not always the primary endpoints. This may limit the strength of the conclusions regarding the effect of JAK inhibitors on these symptoms. Furthermore, the lack of direct (head-to-head) studies comparing specific JAK inhibitors prevents a reliable assessment of potential differences in their efficacy with regard to fatigue and sleep quality.

## 8. Summary

Jak inhibitors, including filgotinib, baricitinib, upadacitinib and tofacitinib, have shown consistent beneficial effects on fatigue in patients with RA, reducing symptom severity and improving patient-reported outcomes. Tofacitinib and upadacitinib have also shown a potential beneficial effect on sleep quality. However, the available evidence remains limited, and the impact of treatment on sleep quality in patients with RA requires further investigation, particularly in the context of modern targeted therapies. 

**Table 1:** Characteristics and results of the main clinical studies included in the review regarding fatigue.

Author, Year	Study Aim	Intervention Groups	Control Groups	Fatigue Measure	Impact on Fatigue
Yang Y. et al., 2021 [44]	To assess the effect of baricitinib on PROs, including fatigue, in patients with moderately to severely active RA and inadequate response to MTX.	Baricitinib 4 mg once daily + MTX (*N* = 145)	Placebo + MTX (*N* = 145)	FACIT-F; Worst Tiredness NRS	Baricitinib rapidly and significantly reduced fatigue vs. placebo. More patients achieved clinically meaningful improvement.
Sholter D. et al., 2022 [45]	To evaluate maintenance of clinically meaningful fatigue improvement with baricitinib 2 mg up to week 24.	RA-BUILD: baricitinib 2 mg once daily (*N* = 229); RA-BEACON: baricitinib 2 mg once daily (*N* = 174)	RA-BUILD: placebo (*N* = 228); RA-BEACON: placebo (*N* = 176)	FACIT-F	Baricitinib increased the proportion of patients achieving and maintaining MCID in fatigue through week 24.
Emery P. et al., 2017 [46]	To evaluate the effect of baricitinib on PROs, including fatigue, in active RA with inadequate response or intolerance to csDMARDs.	Baricitinib 2 mg once daily (*N* = 229); baricitinib 4 mg once daily (*N* = 227); stable csDMARDs permitted	Placebo (N = 228); stable csDMARDs permitted	FACIT-F; Worst Tiredness NRS	Both doses evaluated independently were associated with significant improvements in fatigue outcomes compared with placebo.
Keystone E.C. et al., 2017 [47]	To compare the effect of baricitinib, placebo and adalimumab on PROs, including fatigue, in MTX-IR RA.	Baricitinib 4 mg once daily + csDMARDs, including MTX (*N* = 487)	Placebo + csDMARDs (*N* = 488); adalimumab 40 mg every 2 weeks + csDMARDs (*N* = 330)	FACIT-F; Worst Tiredness NRS	Baricitinib significantly reduced fatigue vs. placebo and showed favourable results vs. adalimumab at selected time points.
Schiff M. et al., 2017 [48]	To assess baricitinib monotherapy and baricitinib + MTX vs. MTX in early or csDMARD-naïve RA.	Baricitinib 4 mg once daily (*N* = 159); baricitinib 4 mg once daily + MTX (*N* = 215)	MTX (*N* = 210)	FACIT-F; Worst Tiredness NRS	Baricitinib, alone or with MTX, reduced fatigue more than MTX. The clearest benefit was observed with monotherapy.
Fautrel B. et al., 2023 [49]	To analyse whether fatigue reduction with baricitinib is mediated by pain reduction and disease activity improvement.	RA-BEAM: baricitinib 4 mg + MTX (*N* = 487); RA-BEACON: baricitinib 4 mg + background therapy (*N* = 177)	RA-BEAM: placebo + MTX (*N* = 488), adalimumab + MTX (*N* = 330); RA-BEACON: placebo + background therapy (*N* = 176)	FACIT-F	Baricitinib reduced fatigue in MTX-IR and bDMARD-IR patients. Fatigue improvement was partly mediated by pain reduction and better disease control.
Wang Y. et al., 2022 [50]	To evaluate the efficacy and safety of filgotinib, including its effect on fatigue, in RA.	Filgotinib 200 mg once daily (*N* = 777); filgotinib 100 mg once daily (*N* = 788)	Placebo (*N* = 781)	FACIT-F	Filgotinib improved fatigue vs. placebo. The effect was stronger and more sustained with 200 mg vs. 100 mg, particularly at week 24.
Bingham C.O. et al., 2022 [51]	To assess the effect of filgotinib on PROs, including fatigue, across three FINCH RA populations.	FINCH 1: filgotinib 200/100 mg + MTX in MTX-IR patients (*N* = 955); FINCH 2: filgotinib 200/100 mg + csDMARDs in bDMARD-IR patients (*N* = 300); FINCH 3: filgotinib 200/100 mg + MTX or filgotinib 200 mg monotherapy in MTX-naïve patients (*N* = 416)	FINCH 1: placebo + MTX, adalimumab + MTX; FINCH 2: placebo + csDMARDs; FINCH 3: MTX	FACIT-F	Filgotinib reduced fatigue across all FINCH populations. In analyses evaluating each dose independently, the 200 mg dose demonstrated a consistent benefit, particularly in FINCH 2 and in the long-term outcomes of FINCH 3.
Strand V. et al., 2020 [52]	To analyse associations between PtGA, pain, function and fatigue in patients treated with tofacitinib.	Tofacitinib 5 mg twice daily + csDMARDs, mainly MTX (*N* = 742)	Placebo + csDMARDs, mainly MTX (*N* = 391)	FACIT-F	Tofacitinib increased the proportion of patients achieving clinically meaningful fatigue improvement by month 3.
Bartlett S.J. et al., 2022 [53]	To assess the effect of tofacitinib on fatigue, sleep, HRQoL and their relationship with disease activity.	Tofacitinib 5 mg twice daily + csDMARDs (*N* = 826); tofacitinib 10 mg twice daily + csDMARDs (*N* = 821)	Placebo + csDMARDs (*N* = 419); adalimumab + csDMARDs (*N* = 199)	FACIT-F	Both tofacitinib doses apprised separately reduced fatigue vs. placebo. The 10 mg dose showed favourable results vs. adalimumab in some analyses. Fatigue correlated with HRQoL and sleep parameters.
Bird P. et al., 2019 [54]	To evaluate tofacitinib efficacy by serological status, including fatigue outcomes.	Tofacitinib 5 mg twice daily (*N* = 1194); tofacitinib 10 mg twice daily (*N* = 1197), analysed by anti-CCP/RF status	Placebo (*N* = 670), analysed by anti-CCP/RF status	FACIT-F	Tofacitinib reduced fatigue vs. placebo across serological subgroups.
Strand V. et al., 2019 [57]	To assess the effect of upadacitinib on PROs, including fatigue, in csDMARD-IR RA.	Upadacitinib 15 mg once daily + csDMARD (*N* = 221); upadacitinib 30 mg once daily + csDMARD (*N* = 219)	Placebo + csDMARD (*N* = 221)	FACIT-F	Upadacitinib at both doses independently reduced fatigue compared to placebo.
Bergman M. et al., 2022 [58]	To compare upadacitinib and abatacept effects on PROs, including fatigue, in bDMARD-IR RA.	Upadacitinib 15 mg once daily + background csDMARDs (*N* = 303)	Abatacept IV according to body weight + background csDMARDs (*N* = 309)	FACIT-F	Both treatments reduced fatigue. Differences between groups were small.
Rubbert-Roth A. et al., 2024 [59]	To evaluate long-term efficacy and safety of upadacitinib up to week 216, including fatigue outcomes.	Continued upadacitinib 15 mg once daily (*N* = 303); switch from abatacept to upadacitinib at week 24 (*N* = 309)	-	FACIT-F	Fatigue improvement was maintained long term in both cohorts.
Strand V. et al., 2021 [62]	To compare upadacitinib, placebo and adalimumab effects on PROs in MTX-IR RA.	Upadacitinib 15 mg once daily + MTX (*N* = 651); adalimumab 40 mg every 2 weeks + MTX (*N* = 327)	Placebo + MTX (*N* = 651)	FACIT-F	Upadacitinib reduced fatigue more than placebo and adalimumab at week 12. Benefits vs. adalimumab were maintained at weeks 26 and 48.

**Table 2:** Characteristics and results of the main REAL-WORLD studies included in the review regarding fatigue.

Author, Year	Study Aim	Intervention Groups	Control Groups	Fatigue Measure	Impact on Fatigue
Harrold L.R. et al., 2023 [55]	To compare abatacept and tofacitinib in CCP-positive RA, including fatigue outcomes.	Abatacept (*N* = 291); tofacitinib (*N* = 291), as monotherapy or with MTX/non-biologic DMARDs	-	Patient-reported fatigue scale	Fatigue decreased in both groups. Abatacept showed a numerically greater reduction, but the difference was not statistically significant.
Taylor J. et al., 2025 [56]	To assess real-world outcomes and PROs after 6 months of upadacitinib treatment.	Upadacitinib (*N* = 93); bDMARD-naïve and bDMARD-IR patients, mostly with background csDMARDs	-	FACIT-F; Fatigue VAS	Upadacitinib produced rapid and sustained fatigue improvement. Clinically meaningful improvement was reached earlier on Fatigue VAS than on FACIT-F.
Baker J.F. et al., 2024 [60]	To evaluate 6- and 12-month outcomes with upadacitinib in moderate to severe RA.	Upadacitinib: 6-month cohort (*N* = 469); 12-month cohort (*N* = 263); subgroups included first-line UPA and TNFi-experienced patients	-	Patient-reported Fatigue VAS	Upadacitinib was associated with significant fatigue reduction. Improvement was greater in first-line UPA patients than in TNFi-experienced patients.
Caporali R. et al., 2024 [61]	To compare treatment strategies after first TNFi failure, including switch to upadacitinib.	Switch from TNFi to upadacitinib (*N* = 261)	Switch to another TNFi (*N* = 128); switch to other MOA therapy (*N* = 114)	Physician-reported fatigue, 4-point scale	Absence of fatigue was more frequent after switch to upadacitinib, but differences vs. other strategies were not statistically significant.

**Table 3:** Characteristics and results of the main clinical studies included in the review on sleep disturbance.

Author, Year	Study Aim	Intervention Groups	Control Groups	Sleep Measure	The Impact of Treatment on Sleep Quality
Bartlett et al., 2022 [53]	To assess the effect of tofacitinib on sleep, fatigue and HRQoL in active RA.	Tofacitinib 5 mg twice daily + csDMARD (*N* = 826); tofacitinib 10 mg twice daily + csDMARD (*N* = 821)	Adalimumab 40 mg + csDMARD (*N* = 199); placebo + csDMARD (*N* = 419)	MOS Sleep Scale	Tofacitinib improved sleep indices from month 1 vs. placebo. Sleep improvement was also greater than with adalimumab.
Strand V. et al., 2017 [69]	To evaluate tofacitinib + csDMARDs on PROs, including sleep quality, in active RA.	Tofacitinib 5 mg twice daily + csDMARD (*N* = 315); tofacitinib 10 mg twice daily + csDMARD (*N* = 318)	Placebo + csDMARD (*N* = 159)	MOS Sleep Scale	Both tofacitinib 5 mg and 10 mg independently improved sleep quality vs. placebo.
Strand, V. et al., 2020 [70]	To assess long-term effects of tofacitinib + MTX on sleep quality over 24 months.	Tofacitinib 5 mg twice daily + MTX (*N* = 321); tofacitinib 10 mg twice daily + MTX (*N* = 316)	Placebo + methotrexate (*N* = 160)	MOS Sleep Scale	Tofacitinib significantly improved sleep quality. Patients switched from placebo achieved similar long-term improvement by month 24.
Strand, V. et al., 2015 [71]	To evaluate tofacitinib + MTX on PROs, including sleep, in TNFi-IR RA.	Tofacitinib 5 mg twice daily + MTX (*N* = 133); tofacitinib 10 mg twice daily + MTX (*N* = 134)	Placebo + MTX (*N* = 132)	MOS Sleep Scale	No statistically significant improvement in overall sleep problems vs. placebo.
Strand, V. et al., 2015 [72]	To assess tofacitinib monotherapy effects on sleep quality in active RA.	Tofacitinib 5 mg twice daily (*N* = 243); tofacitinib 10 mg twice daily (*N* = 245)	Placebo (*N* = 122)	MOS Sleep Scale	Each tofacitinib dose was independently evaluated vs. placebo, showing statistically significant improvements for 10 mg, but not for 5 mg.
Strand, V. et al., 2016 [73]	To compare tofacitinib monotherapy with MTX for sleep outcomes in MTX-naïve RA.	Tofacitinib 5 mg twice daily (*N* = 373); tofacitinib 10 mg twice daily (*N* = 397)	MTX (*N* = 186)	MOS Sleep Scale	Compared separately with MTX tofacitinib 5 mg showed significant improvements in sleep, whereas 10 mg provided no additional benefit.
Strand, V. et al., 2019 [74]	To evaluate upadacitinib effects on insomnia severity in bDMARD-IR RA.	Upadacitinib 15 mg once daily + csDMARDs (*N* = 164); upadacitinib 30 mg once daily + csDMARDs (*N* = 165)	Placebo + csDMARDs (*N* = 169)	Insomnia Severity Index	Each dose was assessed separately vs. placebo: upadacitinib 30 mg significantly improves insomnia severity, while the 15 mg dose showed a numerical, non-significant improvement.

## Data Availability

No new data were created or analyzed in this study. Data sharing is not applicable to this article.
